# Ovarian resection in anti-N-methyl-D-aspartate receptor encephalitis: A comparison of surgical approaches

**DOI:** 10.3389/fneur.2022.1043785

**Published:** 2022-11-17

**Authors:** Yajur Iyengar, Julien Hébert, Seth A. Climans, Alexandra Muccilli, Sydney Lee, Abhilasha P. Boruah, Kiran T. Thakur, Jonathon Solnik, Richard A. Wennberg, Gregory S. Day, David F. Tang-Wai

**Affiliations:** ^1^Department of Medicine, Temerty Faculty of Medicine, University of Toronto, Toronto, ON, Canada; ^2^Division of Neurology, Department of Medicine, University of Toronto, Toronto, ON, Canada; ^3^Comprehensive Epilepsy Center, Columbia University Irving Medical Center - New York Presbyterian Hospital, New York, NY, United States; ^4^Department of Medical Oncology and Hematology, Princess Margaret Cancer Centre - University Health Network, New York, NY, United States; ^5^Multiple Sclerosis Clinic, St. Michael's Hospital, Toronto, ON, Canada; ^6^Division of Hospitalist and Critical Care Neurology, Columbia University Irving Medical Center - New York Presbyterian Hospital, New York, NY, Canada; ^7^Department of Obstetrics and Gynaecology, Mount Sinai Hospital, Toronto, ON, Canada; ^8^Epilepsy Clinic, Toronto Western Hospital - University Health Network, Toronto, ON, Canada; ^9^Department of Neurology, Mayo Clinic Florida, Jacksonville, FL, United States; ^10^Memory Clinic, Toronto Western Hospital - University Health Network, Toronto, ON, Canada

**Keywords:** anti-NMDAR Encephalitis, ovarian resection, functional outcome, retrospective cohort, meta–analysis

## Abstract

**Background:**

For patients with anti-N-methyl-D-aspartate receptor encephalitis (NMDARE) and ovarian teratoma, “conservative” surgical approaches (complete or partial unilateral oophorectomy or bilateral partial oophorectomies) are associated with clinical improvement. “Aggressive” ovarian resections (complete bilateral oophorectomy or “blind” ovarian resections without pre-operative evidence of teratoma) are also reported, although the evidence supporting these approaches is unclear.

**Objective:**

To compare the one-year functional outcomes of patients with NMDARE who underwent conservative vs. aggressive ovarian resections.

**Methods:**

Patients with NMDARE undergoing ovarian resection between January 1st, 2012 and December 31st, 2021 were retrospectively identified from three North American tertiary care centers. Primary outcome was a modified Rankin Scale score of 0–2 one year after ovarian resection. Fisher exact and Wilcoxon rank sum tests were used to compare demographic features, disease characteristics, and functional outcomes between the two surgical groups. A fixed-effects meta-analysis of studies reporting functional outcomes based on surgical approach was also performed.

**Results:**

Twenty-three patients were included. Eight underwent aggressive surgical management. There was a non-significant trend toward an association between aggressive surgical management and younger age-at-onset, higher baseline disease severity, and longer delays to treatment. There was no difference between “aggressive” (3/8, 38%) and “conservative” (11/15, 73%) management groups in achieving the primary outcome (OR_95%_ = <0.1–1.9; *p* = 0.18). Findings were similar when considering data from 52 patients in two published studies (RR = 0.74; CI_95%_ = 0.48–1.13; *p* = 0.16).

**Conclusions:**

Aggressive ovarian resection was not associated with improved outcomes in patients with NMDARE in this series. Group differences may have contributed, recognizing that patients who underwent aggressive resection tended to be sicker, with procedures performed later in the disease course. Based on available evidence, we advocate for function-sparing resection in patients with imaging-confirmed/suspected teratoma, and repeated multi-modal imaging in at-risk patients with NMDARE refractory to conventional treatment.

## Introduction

Ovarian teratomas including neurological tissue (1, 2) are identified in ~20% of patients with anti-N-Methyl-D-Aspartate Receptor Encephalitis (NMDARE) (3, 4). Early teratoma resection is an important part of the treatment plan in these patients and is associated with improved clinical outcomes, shorter time to recovery, and lower risk of disease relapse (5–10). In most cases, unilateral ovarian resections are performed in patients with ovarian lesions identified on appropriate imaging (11, 12). In patients with suspected bilateral ovarian teratomas, “partial” bilateral oophorectomy may be undertaken to resect tumor tissue while preserving ovarian function (12–18). Despite a paucity of evidence to support their use, “blind” ovarian resections (i.e., resections performed without pre-operative imaging evidence of teratoma) and bilateral “full” oophorectomies have been performed on patients with refractory NMDARE. Proponents of these “aggressive” surgical approaches argue that the increased risks of surgical complications, infertility, and iatrogenic premature menopause are justified in refractory cases, citing reports of occult imaging-negative microscopic teratomas found only on histopathology (19, 20). However, this rationale minimizes the potential for bias in publication of cases in which a microscopic teratoma *is* detected following resection, underemphasizes the risk of long-term complications following ovarian resection in surviving patients, and overlooks an expanding body of literature emphasizing that most NMDARE cases are not associated with ovarian teratomas (3, 4).

Quantitative data on the relative risks and benefits of different approaches to ovarian teratoma resection in NMDARE is needed to support clinical decision making. The need for quantitative data is even more apparent given the ethical challenges inherent in the decision to perform a surgery with potential long-term consequences on physical health and fertility in patients who are generally unable to comprehend the need for the procedure, inherent risks, or alternatives. In response to this need, we compared the one-year functional and neurological outcomes of patients with NMDARE who underwent “aggressive” surgical management with those of patients for whom a more “conservative” surgical approach was undertaken.

## Materials and methods

### Patient selection and data collection

Data from patients with definite NMDARE, who underwent ovarian resection between January 1st 2012 and December 31st 2021 at one of the three participating tertiary care centers (Toronto Western Hospital—University Health Network [Toronto, ON, Canada], Washington University in St. Louis [St. Louis, MO, USA], and Columbia University Irving Medical Center [New York City, NY, USA]) were included in this study. All patients met existing clinical criteria for definite NMDARE (21). The following was collected through a retrospective chart review: demographics, ovarian imaging type and findings, immunomodulatory therapies received, baseline EEG and MRI findings, ovarian surgical management (See *Exposures*), teratoma histopathology, and neurological and functional outcome (See *Outcome Measures*). Patients without information on surgical technique used or one-year functional outcomes were excluded.

### Exposures

**Figure 1:**
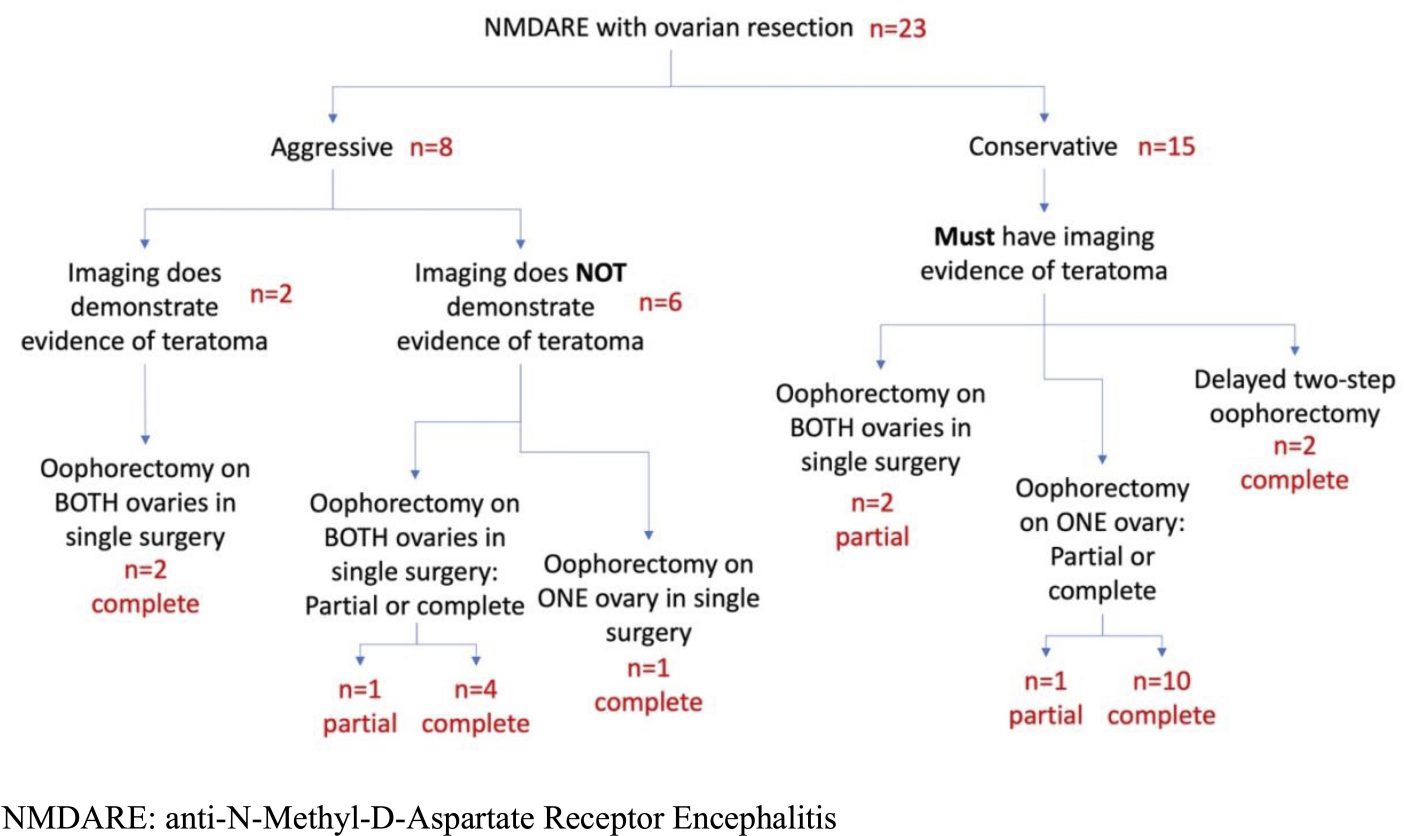
Patient Flowchart. NMDARE, anti-N-methyl-D-aspartate receptor encephalitis.

Approaches to ovarian resection were dichotomized as “aggressive” or “conservative” (Figure 1). We defined an “aggressive” surgical approach as either, a) bilateral “full” oophorectomy; *or*, b) ovarian resection performed without pre-operative imaging evidence of teratoma (i.e., “blind” resection). Unilateral “partial” or “full” oophorectomies, and bilateral “partial” oophorectomies were considered “conservative” if teratomas were suspected based on pre-operative ovarian imaging. In patients who underwent a unilateral “full” oophorectomy followed by complete resection of the contralateral ovary due to lack of clinical improvement or relapse (“delayed” bilateral oophorectomy), only the first unilateral resection was considered.

### Outcome measures

The primary outcome was “good” one-year functional status, defined as an mRS score of 0–2 at one-year follow-up (8, 22). Secondary outcomes were early clinical improvement 4-weeks after treatment initiation, seizure burden reduction of >50% at one-year, and improvement in total Montreal Cognitive Assessment (MoCA) (23) score at one-year. Due to the retrospective nature of the study, the “one-year” time requirement was established at any follow-up that occurred between 9 and 15 months after treatment initiation.

### Confounding variables

We used the anti-NMDAR Encephalitis One-Year Functional Status (NEOS) to account for differing degrees of baseline disease severity among the two surgical management approach groups, whereby higher NEOS scores signify increased disease severity (24). Other confounding variables taken into account were patient age at symptom onset, time from symptom onset to surgery, time from symptom onset to immunotherapy, and length of intensive care unit (ICU) stay. We used Fisher exact and Wilcoxon rank sum tests to compare, respectively, categorical and continuous variables between the different surgical approaches.

### Meta-analysis

We searched the Pubmed Database for English literature containing the terms “N-Methyl-D-Aspartate receptor encephalitis,” “NMDA Encephalitis,” and “ovarian teratoma.” We excluded studies which reported on encephalitis other than NMDARE, studies that included only males, case reports, and review articles. This was independently reviewed by two co-authors (YI, JH). We then closely analyzed the studies thus screened and excluded those for which no patients underwent ovarian resection, no follow-up information was provided at one-year, no surgical data was available, or no patients underwent aggressive surgical resection. In the remaining studies, if patient information stratified by surgical management approach was not available, we contacted the authors (25). We then performed a fixed-effects meta-analysis integrating the results from our study with those identified through our literature review (26).

### Power calculations

For power analysis, we used a bootstrap method with a putative Fisher exact test, using alpha = 0.05 and 10,000 simulations (27). For estimations of effect size, we considered a fictional baseline patient sample with severe baseline impairment, defined as a NEOS score of 4–5 (i.e., 31% chance of achieving the primary outcome). We hypothesized that the more aggressive treatments were performed on patients with more severe baseline disease severity. A “large” effect size of aggressive surgical management was defined as equivalent to a 4-point NEOS score reduction, which translates into a 97% change of achieving “good” functional status at one-year; a “moderate” effect size was equivalent to a 3-point NEOS score reduction (i.e., 85% chance of achieving a “good” primary outcome); and a “small” effect as a 1-point reduction on the NEOS score (i.e., 75% chance of achieving a “good” primary outcome) (24).

### Statistical analysis

R Statistical Software version 4.1.2 (The R Foundation for Statistical Computing, 2021) was used for statistical analysis (statistical coding available upon request).

### Ethical conduct of research

This research project received approval from each institution's Research Ethics Board.

## Results

After excluding two patients due to missing data, 23 patients met inclusion criteria (Figure 1). All had IgG autoantibodies targeting NMDAR in CSF (“definite” NMDAR encephalitis). Most of our cohort (96%) was of child-bearing age, with presentations dominated by cognitive impairment (19/22, 86%) or psychiatric symptoms (21/23, 91%; Table 1). Movement disorders (14/23, 61%), seizures (15/23, 65%), and dysautonomia (14/21, 67%) were also prevalent. Of 21 patients with available MRI imaging (91%), five (24%) had temporal lobe FLAIR signal changes. Of 22 patients with CSF results available (96%), 10 (45%) had > 20 WBCs/dL. The eight patients (35%) who underwent “aggressive” surgical management tended to be younger, have longer delays from symptom onset to surgical and immunosuppressive treatment, and to have higher baseline NEOS scores (Table 1). While almost all patients in both groups received a combination of intravenous steroids and intravenous immunoglobulin, patients in the aggressive surgical management group were more likely to undergo plasmapheresis. No patients in the aggressive management group received rituximab.

**Table 1:** Demographic, disease, treatment, and outcome characteristics by ovarian surgical management approach.

	**Aggressive Approach**	**Conservative Approach**	**Combined** **Sample**	***p*–value**
Sample size, *n (%)*	8 (35)	15 (65)	23 (100)	NA
**Demographics**				
Age, median (range), years	22 (20–73)	28 (19–47)	26 (19–73)	0.58
Partial oophorectomy, *n (%)*	1 (12)	2 (13)	3 (13)	>0.99
Bilateral intervention at onset, *n (%)*	6 (75)	2 (13)	8 (35)	< 0.01^**^
**Disease Characteristics**				
Teratoma on pre–operative imaging, *n (%)*	2 (25)	15 (100)	17 (74)	< 0.01^**^
Teratoma found on pathology, *n (%)*	3 (38)	10 (80)	15 (65)	0.14
NEOS Score, median (range)	3 (2–4)	2 (0–4)	3 (0–4)	0.12
**Treatment**				
Symptom onset to surgery, median (range), days	72 (16–210)	31 (11–129)	33 (11–210)	0.40
Intravenous steroids, *n (%)*	8 (100)	14 (93)	22 (96)	>0.99
Intravenous Immunoglobulin, *n (%)*	7 (88)	13 (87)	20 (87)	>0.99
Plasmapheresis, *n (%)*	7 (88)	4 (27)	11 (48)	< 0.01
Rituximab, *n (%)*	0 (0)	4 (27)	4 (17)	0.26
Admitted to ICU, *n (%)*	8 (100)	13 (87)	21 (91)	0.53
Length of ICU Stay, median (range), days	28 (3–300)	19 (1–190)	25 (1–300)	0.73
**Outcomes**				
Good one–year functional outcome, *n (%)*	3 (38)	11 (73)	14 (61)	0.18
Clinical improvement at 4–weeks, *n (%)*	1 (13)	8 (53)	9 (39)	0.09

^*^p < 0.05;

** p < 0.001;

ICU, Intensive Care Unit; NA, Not available; NEOS: anti–NMDAR Encephalitis One–Year Functional Status.

Twenty-one patients (91%) underwent pre-operative ovarian imaging. Ultrasound was the most used imaging modality (*n* = 13, 62%), followed by MRI (*n* = 5, 22%), CT (*n* = 4, 18%), and FDG-PET (*n* = 2, 9%). Multiple modalities were used in three patients (13%) (Table 2). Three out of seventeen patients (18%) with pre-operative evidence of possible teratomas did not have a teratoma on pathology (“false positive”) and instead were found to have an epidermal inclusion cyst, a hemorrhagic corpus luteum, and a tubo-ovarian abscess (Table 2; Cases 21, 22, 26). A teratoma was identified on pathology in 1/5 patients (20%) without pre-operative evidence of a teratoma (“false negative”; Table 2, Case 12).

**Table 2:** One–year functional outcomes by ovarian surgical approach.

**Case**	**Age, years^a^**	**NEOS Score**	**Ovarian Imaging**		**Oophorectomy**	**Ovarian Pathology^b^**	**mRS^c^**
			**Type**	**Findings**			
**Aggressive surgical management**							
6	21	3	MRI	Bilateral PCOS	Laparoscopic bilateral full	Bilateral PCOS	1
8	31	3	US	Right teratoma	Laparoscopic bilateral full	Right teratoma, left normal	3
12	23	3	MRI	Normal Ovaries	Laparoscopic bilateral full	Right teratoma, left normal	3
13	20	3	PET	Normal Ovaries	Open bilateral full	*Not available*	2
16	21	2	MRI	Normal Ovaries	Laparoscopic full left	Left normal	2
19	73	4	CT,US	Left Fibroma	Laparoscopic bilateral full	Left benign fibroma	6
20	33	3	US	Bilateral teratoma	Laparoscopic bilateral full	Bilateral teratoma	3
25	21	4	US,CT,PET	Normal ovaries	Bilateral open partial^d^	Bilateral normal	3
**Conservative surgical management**							
2	19	1	US	Left teratoma	Open left partial	Left teratoma	1
3	24	1	US	Right teratoma	Open right full^d^	Right teratoma	0
4	38	3	US	Left teratoma	Laparoscopic left full	Left teratoma	4
9	28	2	NA	Right teratoma	Laparoscopic right full^d^	Right teratoma	1
10	30	0	US	Bilateral teratoma	Laparoscopic bilateral partial	Bilateral teratoma	0
11	29	3	MRI	Right teratoma	Open right full	Right teratoma	0
14	21	4	US	Right teratoma	Laparoscopic right full^d^	Right teratoma	4
15	20	4	US,MRI	Bilateral cyst	Laparoscopic left full/right partial	Bilateral teratoma	1
17	23	1	US	Left teratoma	Laparoscopic left full	Left teratoma	0
18	33	2	CT	Right teratoma	Laparoscopic right full^d^	Right teratoma	0
21	31	4	US	Left teratoma	Laparoscopic left full	Left epidermal inclusion cyst	5
22	36	3	US	Right calcification	Laparoscopic right full	Right tubo–ovarian abscess	2
23	21	2	NA	Right teratoma	Laparoscopic right full	Right teratoma	3
24	26	3	US	Right teratoma	Laparoscopic right full	Right teratoma	0
26	47	1	CT	Right calcification	Laparoscopic right full	Hemorrhagic corpus luteum	1

a Age at symptom–onset.

b Teratomas are all mature.

c at one–year follow–up.

d Subsequently had delayed contralateral full oophorectomy due to lack of clinical improvement or relapse. CT, Computed Tomography; MRI, Magnetic Resonance Imaging; mRS, modified Rankin Scale; NA, Not Available; NEOS, anti–NMDAR Encephalitis One–Year Functional Status; PCOS, Polycystic Ovarian Syndrome; PET, Positron Emission Tomography; US, Ultrasound.

Twelve of eighteen patients (67%) who underwent laparoscopic surgery had findings consistent with a teratoma on histopathology compared with 3/5 (60%) of the patients who underwent an abdominal laparotomy (open resection; *p* < 0.99). No teratoma was identified in either of the 2 patients who underwent laparotomy following negative pre-operative imaging (Table 2).

Fourteen patients (61%) achieved a “good” one-year functional outcome. Patients in the aggressive surgical management group tended to have worse one-year functional outcomes and lower rates of clinical improvements 4 weeks after receiving treatment (Table 2), although the relationship did not reach predetermined levels of statistical significance. All 15 patients who had seizures at onset achieved >50% reduction in seizure frequency at one-year. Only four patients had MoCA scores available at one year (scores = 18, 23, 26, and 29).

Five patients (22%) underwent delayed contralateral ovarian resection a median of 4.0 years (range, 1–11) following initial resection. In all but one case, the delayed contralateral resection was performed due to a clinical relapse. Case 25 was incidentally found to have an ovarian teratoma as part of a work-up for abdominal pain 11 years after the initial resection (prior pelvic imaging negative). During those 11 years, the patient only achieved partial clinical recovery (mRS = 3).

**Figure 2:**
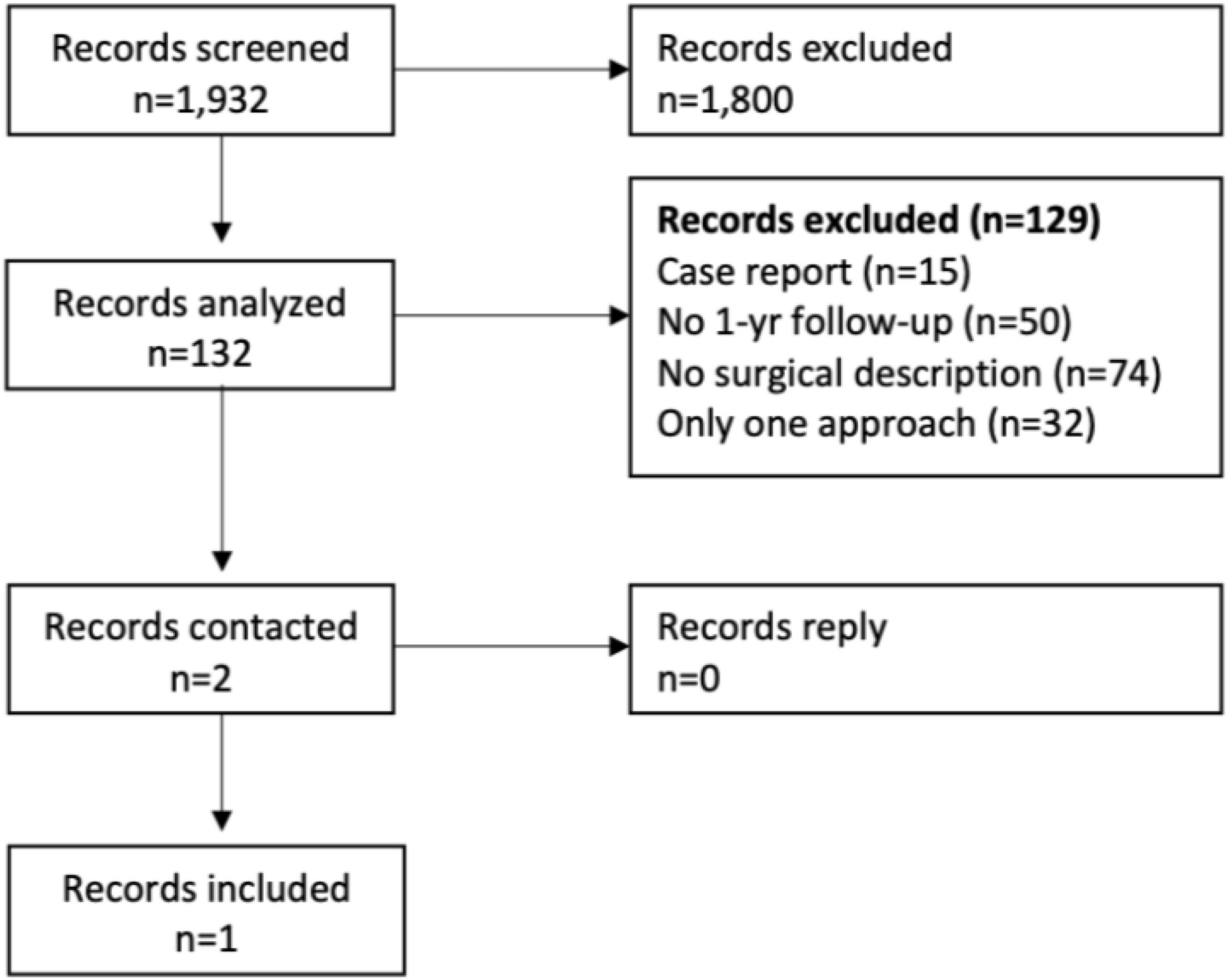
Meta-analysis PRISMA flow diagram. Methyl-D-aspartate receptor encephalitis.

For the meta-analysis, only one study met our inclusion criteria (11) (Figure 2, Table 3). Indicators of heterogeneity were high: τ = 0.37, I^2^ = 53.6% [0.0–88.6%], H = 1.47 [1.00–2.96] and Qdf = 2.16. No difference in the one-year functional outcomes of the two ovarian surgical approach groups was detected after combining studies (RR = 0.74; CI_95%_ = 0.48–1.13; *p* = 0.16; Table 4).

**Table 3:** Reported ovarian surgical approaches and outcomes in patients with N–methyl–D–aspartate encephalitis.

	**Sample Size, *n* (%)**	**Unilateral resection at onset, *n* (%)**	**Bilateral resection at onset, *n* (%)**	**Imaging–negative resection, *n* (%)**	**Outcome measures**
Dai et al. (11)	29 (100)	25 (86)	4 (14)	0 (0)	12–month mRS
El Hanna et al. (18)	1 (100)	1 (100)	0 (0)	0 (0)	7–month Clinical Improvement
Jiang et al. (17)	20 (100)	20 (100)	0 (0)	0 (0)	3–, 6– and 12–month mRS
Lesher et al. (15)	1 (100)	1 (100)	0 (0)	0 (0)	1–month Clinical improvement
Seki et al. (14)	1 (100)	1 (100)	0 (0)	0 (0)	4–month MMSE
Zhang et al. (16)	26 (100)	NA	NA	NA	3–, 6– and 12 –month modified Rankin Scale

MMSE, Mini–Mental State Examination; mRS, Modified Rankin Scale; NA, not available.

**Table 4:** Fixed–effects meta–analysis of 1–year functional outcomes by surgical ovarian management approach.

	**Iyengar et al., this study**	**Dai et al. (11)**	**Combined cohorts**
Sample size, *n* (%)	23 (44)	29 (56)	52 (100)
Aggressive surgical approach, *n* (%)	8 (67)	4 (33)	12 (100)
Conservative surgical approach, *n* (%)	15 (38)	25 (63)	40 (100)
Good functional outcome, *n* (%)	14 (33)	28 (67)	42 (100)
Relative risk of achieving good outcome with aggressive approach (CI_95%_)	0.51	1.04	0.74
	(0.20–1.32)	(0.96–1.13)	(0.48–1.13)

CI, Confidence Interval.

The power of our retrospective cohort to detect a large, medium, or small effect size of aggressive surgical management on the primary outcome was 88, 65, and 45%, respectively. With the meta-analysis, power was increased to >99, 94, and 79% for large, moderate, and small effect sizes, respectively.

## Discussion

Aggressive ovarian resection approaches did not confer improved one-year functional outcomes compared to more conservative approaches in patients with NMDARE. Patients who underwent aggressive surgical resections tended to have higher baseline NEOS scores—a correlate for greater disease severity (24)—and younger age at symptom onset. These findings suggest that aggressive approaches were favored in patients for whom a treatment response was felt to be more urgently needed. The large proportion of laparoscopic surgical methods used in our cohort supports this observation and highlights the need for further study regarding the role of explorative surgery in challenging cases. The importance of judiciously selecting an ovarian resection approach in this patient population is highlighted by the predominance of patients who were of child-bearing age and therefore at risk of surgically induced infertility and menopause.

Improved imaging techniques to better identify teratomas in this patient population could lead to earlier, more circumscribed surgical interventions that might improve long-term outcomes, while minimizing peri-procedural and post-procedural risks, including infertility and early menopause. Variability in imaging techniques, such as operator dependence in the case of ultrasound or center-dependent protocols for MRI, may lead to inconsistent imaging performance. Our single case of “false negative” pre-operative ovarian imaging (Case 12) only underwent abdominal/pelvic MRI. Use of a combination of ovarian imaging techniques may increase our ability to accurately identify a teratoma in this patient population. Multimodal imaging could be considered when suspicion for a teratoma remains high. Additionally, drawing from the experience of the patients in our cohort, we would also recommend periodic ovarian imaging in patients with NMDARE who fail to respond to immunosuppressive treatment or present with NMDARE relapse. Additionally, escalating treatment with other immunosuppressive agents, such as rituximab, may also prove beneficial. Emerging evidence suggests that novel NMDARE biomarkers may identify patients with NMDARE who harbor an ovarian teratoma (28, 29). Nonetheless, the benefits of earlier ovarian teratoma detection are likely to apply to a minority of patients with NMDARE given the low prevalence of teratoma found on pathology in our cohort of patients who underwent ovarian resection without pre-operative imaging evidence of teratoma, and the disproportionate number of patients with NMDARE not associated with a teratoma (3, 4).

### Limitations

Our study was sufficiently powered to detect a large or even moderate effect of such aggressive approaches on long-term functional outcomes, in part due to the inclusion of data from three different centers in two different countries, and integration of data from a comparable study into a meta-analysis. In other words, the study does not provide evidence to reject the null hypothesis that there is no difference in functional outcomes between the two treatment groups. This might be due to either a true lack of difference in improvement or from a failure to detect more modest improvements in functional outcomes due to small sample size. In addition, our meta-analysis only yielded a single study to be included, with resulting high heterogeneity in the results. Future studies incorporating greater numbers of patients across multiple sites are needed to control for the contributions of baseline severity and other differences in management approaches to outcomes, and for adjusting the effects of confounding factors. We were additionally unable to conduct a survival analysis enabling us to compare the outcomes longitudinally or after the one-year follow-up period.

## Conclusions

“Aggressive” approaches to ovarian resection were not associated with improved one-year functional outcomes in NMDARE patients included in this study. Accordingly, we advocate for targeted function-sparing ovarian resections in patients with NMDARE with suspected teratoma. In patients with NMDARE without evidence of ovarian teratoma who are refractory to immunosuppressive treatments we favor repeated multi-modal ovarian imaging in parallel with escalation of immunosuppressive and symptomatic treatment, rather than “blind” ovarian resections. Clinicians who advocate for aggressive bilateral ovarian resection should clearly inform patient and caregivers that this approach has not been shown to be associated with improved outcomes at one-year follow-up. Larger studies are needed to replicate these results and to inform the optimal approach to teratoma surveillance in patients with NMDARE.

## Data availability statement

The original contributions presented in the study are included in the article/supplementary material, further inquiries can be directed to the corresponding authors.

## Ethics statement

The studies involving human participants were reviewed and approved by University Health Network Research Ethics Board, Washington University in St. Louis Research Ethics Board, and Columbia University Irving Medical Center Research Ethics Board. Written informed consent for participation was not required for this study in accordance with the National Legislation and the Institutional Requirements.

## Author contributions

YI was involved in manuscript writing and editing, patient chart review, and meta-analyses. DT-W, KT, SL, GD, AM, and SC were involved in manuscript editing and patient chart review. RW, AB, and JS were involved in manuscript editing. JH was involved in manuscript writing and editing, statistical analyses, and meta-analyses. All authors contributed to the article and approved the submitted version.

## Funding

GD was supported by a career-development award from NIH/NIA (K23AG064029). KT was supported by career-development award from NIH/NINDS K23NS105935. JH received salary-support during his work on this study through a grant from the American Epilepsy Society. Patient enrollment and data collection at Washington University in St. Louis was supported by NIH/NIA (K23AG064029; GD). Patient enrollment and data collection at Columbia University Irving Medical Center-New York Presbyterian Hospital was supported by NIH/NINDS K23NS105935 (PI KT).

## Conflict of interest

The authors declare that the research was conducted in the absence of any commercial or financial relationships that could be construed as a potential conflict of interest.

## Publisher's note

All claims expressed in this article are solely those of the authors and do not necessarily represent those of their affiliated organizations, or those of the publisher, the editors and the reviewers. Any product that may be evaluated in this article, or claim that may be made by its manufacturer, is not guaranteed or endorsed by the publisher.
